# Supplementary material for: A drop dispenser for simplifying on-farm detection of foodborne pathogens
Source: PLoS One. 2024 Dec 31;19(12):e0315444. doi: 10.1371/journal.pone.0315444 (PMC11687705; doi:10.1371/journal.pone.0315444)
Supplement: S1 File — (DOCX) [file pone.0315444.s001.docx]

**Supporting Information**

**for**

**A drop dispenser for simplifying on-farm detection of foodborne pathogens**

Mohsen Ranjbaran^1,2^ , Simerdeep Kaur^1,2^, Jiangshan Wang^1,2^, Bibek Raut^3^, Mohit S. Verma^1,2,3*^

*^1^Department of Agricultural and Biological Engineering, Purdue University, West Lafayette, IN 47906, USA*

*^2^Birck Nanotechnology Center, Purdue University, West Lafayette, IN 47906, USA*

*^3^Weldon School of Biomedical Engineering, Purdue University, West Lafayette, IN 47906, USA*

Corresponding author’s email: [msverma@purdue.edu](mailto:msverma@purdue.edu)

**Supporting Information**

**S1 Fig.** **A comparison between the performance of some commercial pipettors versus our drop dispenser.** All values are in µL.

**S2 Fig**. **A calibration curve to estimate the *E. coli* O157:H7 cell counts from OD_600_ reads.**

**S3 Fig.** **Quantification of the *E. coli* O157:H7 DNA extract using PicoGreen dye.** a) Correlating the Lambda DNA concentration with fluorescence intensity. b) Estimating template DNA concentration.

**S4 Fig.** **A comparison of the LAMP assay results (replicate 1) when using our drop dispenser versus a standard Eppendorf 20-200 µL pipettor.** A yellow color indicates a positive result of the test. The template was STEC O157:H7 DNA extract at various dilutions.

**S5 Fig.** **A comparison of the LAMP assay results (replicate 2) when using our drop dispenser versus a standard Eppendorf 20-200 µL pipettor.** A yellow color indicates a positive result of the test. The template was *E. coli* O157:H7 DNA extract at various dilutions.

**S6 Fig.** **A comparison of the LAMP assay results (replicate 3) when using our drop dispenser versus a standard Eppendorf 20-200 µL pipettor.** A yellow color indicates a positive result of the test. The template was *E. coli* O157:H7 DNA extract at various dilutions.

**S7 Fig.** **A comparison of the whole-cell LAMP assay results when using our drop dispenser versus a standard Eppendorf 20-200 µL pipettor.** A yellow color indicates a positive result of the test. The template was *E. coli* O157:H7 cells at various dilutions.

**S8 Fig.** **Results of LOD tests for whole-cell LAMP assays using *E. coli* O157:H7 cells at various dilutions.** A yellow color indicates a positive result of the test. All liquid handling were performed using a standard Eppendorf 20-200 µL pipettor.

**S1 Table.** **LAMP primer set used to detect *E. coli* O157:H7 by targeting *stxI* gene.**

**S2 Table. Performance of the drop dispensers for several days after surface treatment with plasma and PEG 400.**

**S3 Table.** **Bill of materials for colorimetric LAMP using drop dispensers.**

**S1 File. Drop dispenser plunger design file.**

**S2 File. Drop dispenser liquid holders** **design file.**


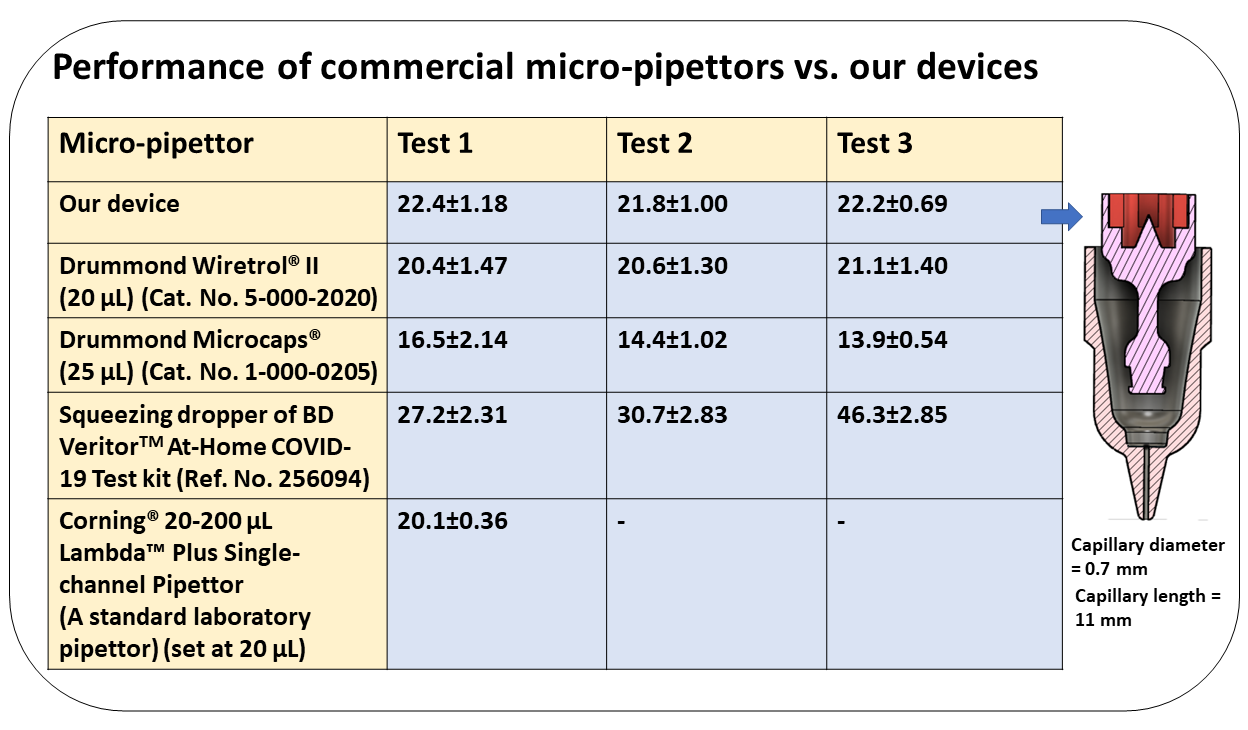


Figure S1. A comparison between the performance of some commercial pipettors versus our drop dispenser. All values are in µL.


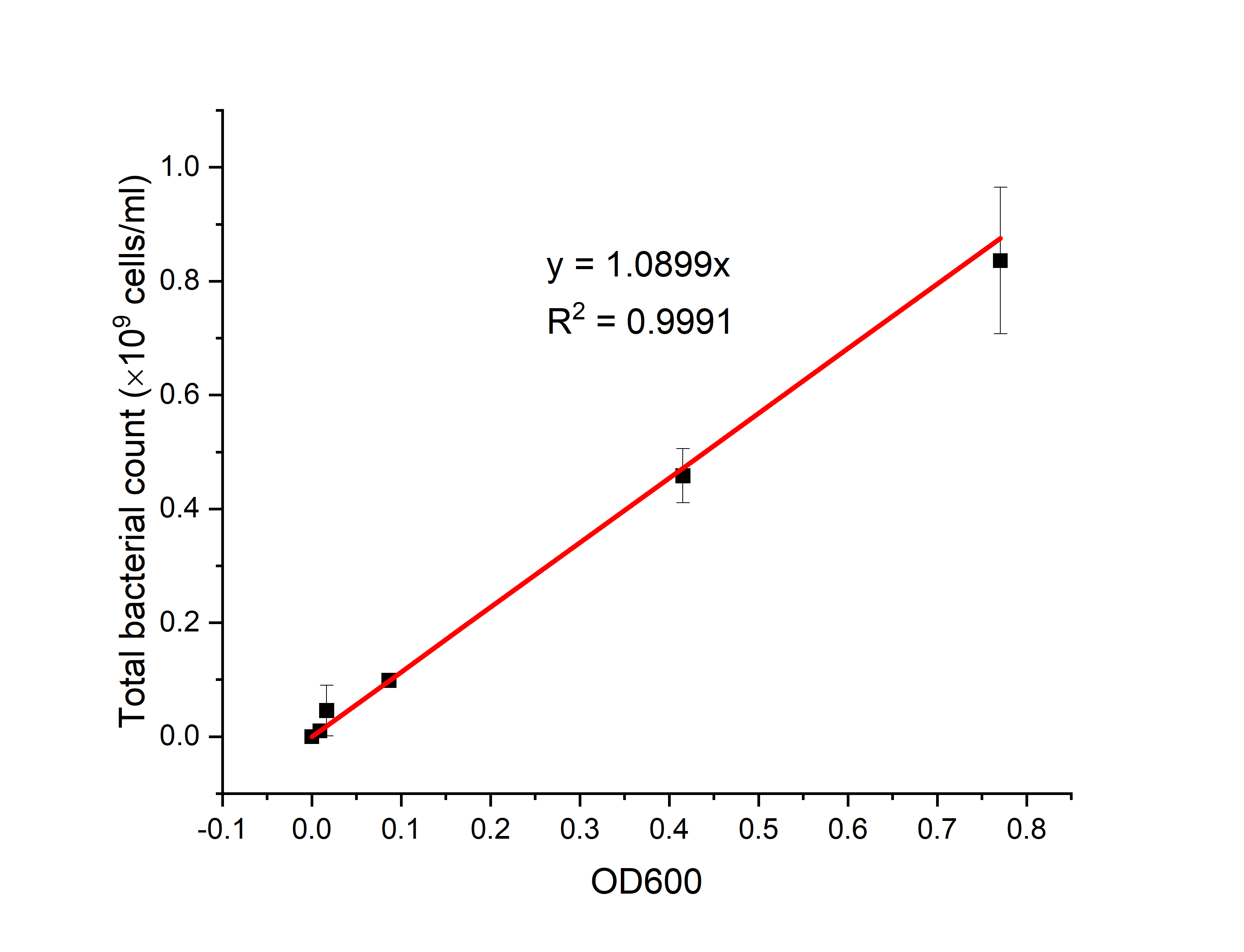


Figure S2. A calibration curve to estimate the *E. coli* O157:H7 cell counts from OD_600_ reads.





Figure S3. Quantification of the *E. coli* O157:H7 DNA extract using PicoGreen dye. a) Correlating the Lambda DNA concentration with fluorescence intensity. b) Estimating template DNA concentration.


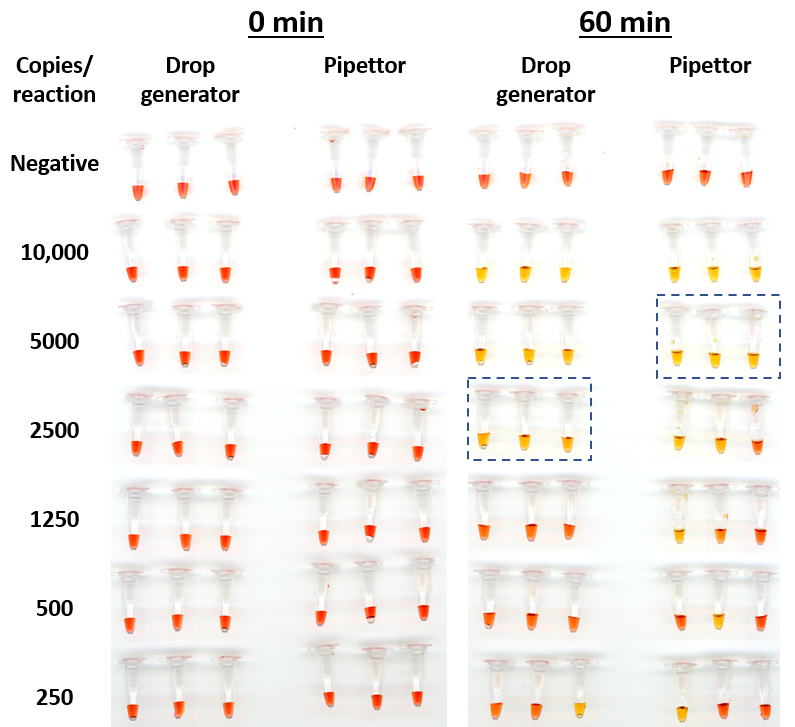


Figure S4. A comparison of the LAMP assay results (replicate 1) when using our drop dispenser versus a standard Eppendorf 20-200 µL pipettor. A yellow color indicates a positive result of the test. The template was *E. coli* O157:H7 DNA extract at various dilutions.


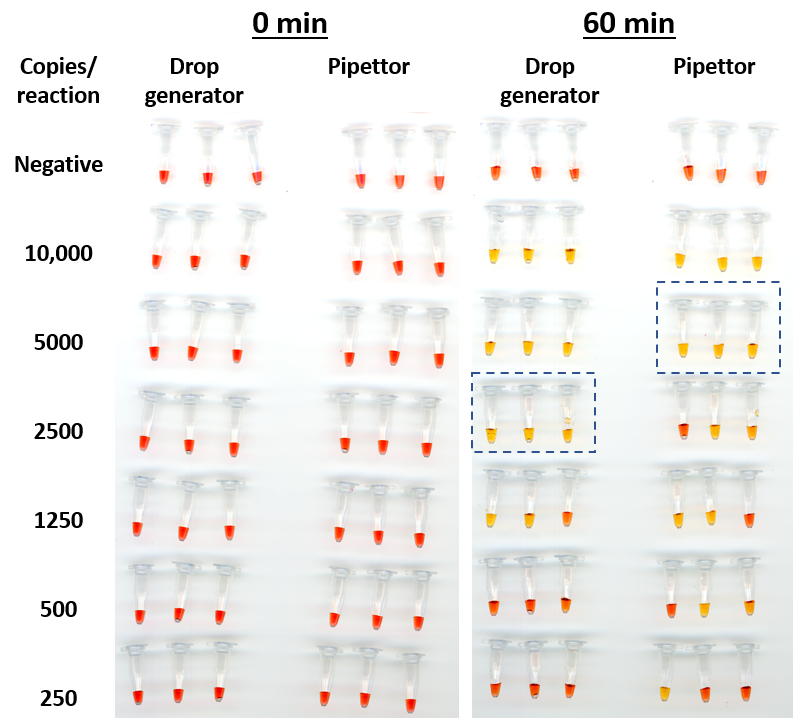


Figure S5. A comparison of the LAMP assay results (replicate 2) when using our drop dispenser versus a standard Eppendorf 20-200 µL pipettor. A yellow color indicates a positive result of the test. The template was *E. coli* O157:H7 DNA extract at various dilutions.


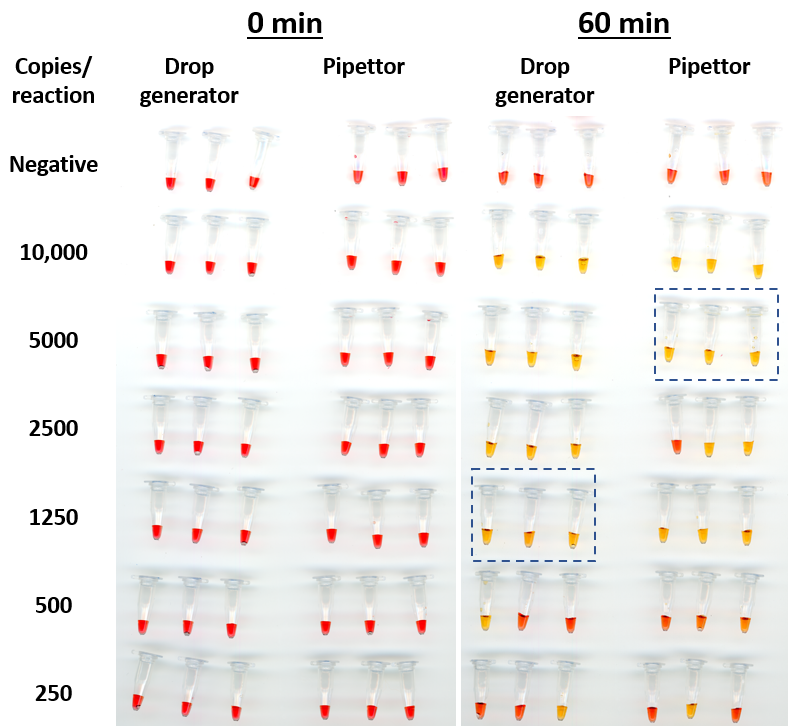


Figure S6. A comparison of the LAMP assay results (replicate 3) when using our drop dispenser versus a standard Eppendorf 20-200 µL pipettor. A yellow color indicates a positive result of the test. The template was *E. coli* O157:H7 DNA extract at various dilutions.


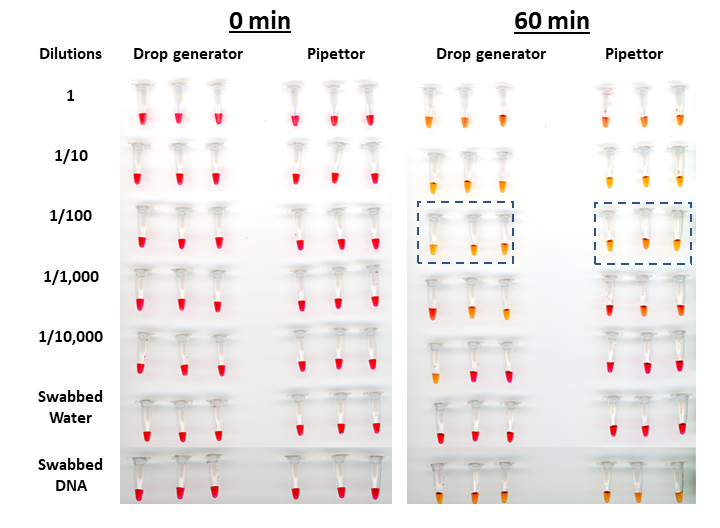


Figure S7. A comparison of the whole-cell LAMP assay results when using our drop dispenser versus a standard Eppendorf 20-200 µL pipettor. A yellow color indicates a positive result of the test. The template was *E. coli* O157:H7 cells at various dilutions.


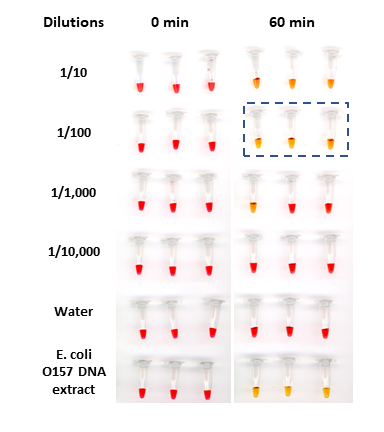


Figure S8. Results of LOD tests for whole-cell LAMP assays using *E. coli* O157:H7 cells at various dilutions. A yellow color indicates a positive result of the test. All liquid handling were performed using a standard Eppendorf 20-200 µL pipettor.

Table S1. LAMP primer set used to detect *E. coli* O157:H7 by targeting *stxI* gene

| Primer name | Sequence (5’-3’) |
| --- | --- |
| EC.stx1-F3 | TGATTTTTCACATGTTACCTTTC |
| EC.stx1-B3 | TAACATCGCTCTTGCCAC |
| EC.stx1-FIP | CCTGCAACACGCTGTAACGTCAGGTACAACAGCGGTTA |
| EC.stx1-BIP | AGTCGTACGGGGATGCAGATAGTGAGGTTCCACTATGC |
| EC.stx1-LF | GTATAGCTACTGTCACCAGACAATG |
| EC.stx1-LB | AAATCGCCATTCGTTGACTACT |

Table S2. Performance of the drop dispensers for several days after surface treatment with plasma and PEG 400.

| Device No. | Day 0 | Day 3 | Day 7 | Day 10 | Day 14 | Day 17 | Day 21 | Day 28 | Day 35 |
| --- | --- | --- | --- | --- | --- | --- | --- | --- | --- |
| Device I | 22.4$\pm$1.18 | $21.4\pm$1.22 | 21.2$\pm$1.14 | 21.5$\pm$  1.21 | 22.4$\pm$  0.80 | 22.2$\pm$  0.88 | 21.3$\pm$  1.40 | 20.7$\pm$  0.82 | 21.3$\pm$  1.07 |
| Device II | 21.8$\pm$1.00 | 21.5$\pm$0.96 | 21.6$\pm$0.85 | 21.1$\pm$  1.30 | 22.3$\pm$  0.89 | 21.3$\pm$  1.17 | 22.3$\pm$  0.91 | 21.5$\pm$  1.08 | Failed^#^ |
| Device III | 22.2$\pm$0.69 | 22.4$\pm$1.14 | 21.5$\pm$1.12 | 21.0$\pm$  1.22 | 21.4$\pm$  1.06 | 21.4$\pm$  1.50 | 22.2$\pm$  0.91 | 22.0$\pm$  1.09 | Failed^#^ |
| Device IV | 21.9$\pm$1.23 | 22.0$\pm$1.12 | 21.09$\pm$1.04 | 22.1$\pm$  0.97 | 22.5$\pm$  0.85 | 22.6$\pm$  1.08 | 22.6$\pm$  1.16 | 22.2$\pm$  1.14 | Failed^#^ |
| Device V | 20.9$\pm$0.84 | 20.9$\pm$0.88 | 20.8$\pm$1.11 | 21.3$\pm$  1.22 | 22.3$\pm$  0.88 | 21.4$\pm$  0.98 | 22.3$\pm$  1.13 | 22.2$\pm$  1.12 | Failed^#^ |
| Device VI | 21.6$\pm$0.96 | 21.4$\pm$1.08 | 22.2$\pm$0.96 | 22.7$\pm$  0.89 | 23.0$\pm$  0.76 | 22.3$\pm$  1.03 | 23.1$\pm$  0.76 | 22.0$\pm$  1.07 | Failed^#^ |

# Devices failed due to uncontrolled dripping after several uses.

Table S3. Bill of materials for colorimetric LAMP using drop dispensers (prices in USD)

| **Item** | **Source** | **Unit price** | **Amount of use per test** | **Price per test** |
| --- | --- | --- | --- | --- |
| **Droplet dispenser** | | | | |
| High Temp V2 resin | Formlabs, RS-F2-HTAM-02 | $199/1 L | 10 mL | $1.99 |
| O-ring | Helipal, Airy-Acc-Oring-2.5×6mm | $2.9/40 pieces | 2 pieces | $0.15 |
| **Sub-total** |  |  |  | **$2.14 (55 %)** |
| **LAMP assay** | | | | |
| Magnesium Sulfate | Sigma-Aldrich, M2773 | $68.20/500 g | 0.0002 g | $0.00 |
| Potassium Chloride | Sigma-Aldrich, P9541 | $47.80/500 g | 0.0004 g | $0.00 |
| Antarctic Thermolabile UDG | New England Biolabs, M0372S | $80.00/100 U | 0.0875 U | $0.07 |
| dNTPs | Fisher Scientific, FERR0182 | $634.50/4 mL | 0.0014 mL | $0.22 |
| dUTP | Fisher Scientific, FERR0133 | $80.70/250 µL | 0.0875 µL | $0.03 |
| Phenol Red | Sigma-Aldrich, P3532 | $105.00/25 g | 0.0094 g | $0.04 |
| Betaine | Sigma-Aldrich, B0300-5VL | $103.00/7.5 mL | 0.0001 mL | $0.00 |
| EC.stx1 Primer mix | Life Technologies, N/A | $68.44/12500 reactions | 1 reaction | $0.01 |
| Warmstart *Bst* 2.0 DNA Polymerase | New England Biolabs, M0537M | $296.00 /0.067 mL | 0.0001 mL | $0.44 |
| **Sub-total** |  |  |  | **$0.81 (21 %)** |
| **Sample collection** | | | | |
| BD BBL Dacron Polyester-Tipped Swabs | BD, 263000 | $189.5/500 swabs | 1 swab | $0.85 |
| Transparency film | Apollo, 617993 | $32.99/100 sheets | 0.3 sheet | $0.10 |
| **Sub-total** |  |  |  | **$0.95 (24 %)** |
|  | | | | |
| **Total** |  |  |  | **$3.9** |


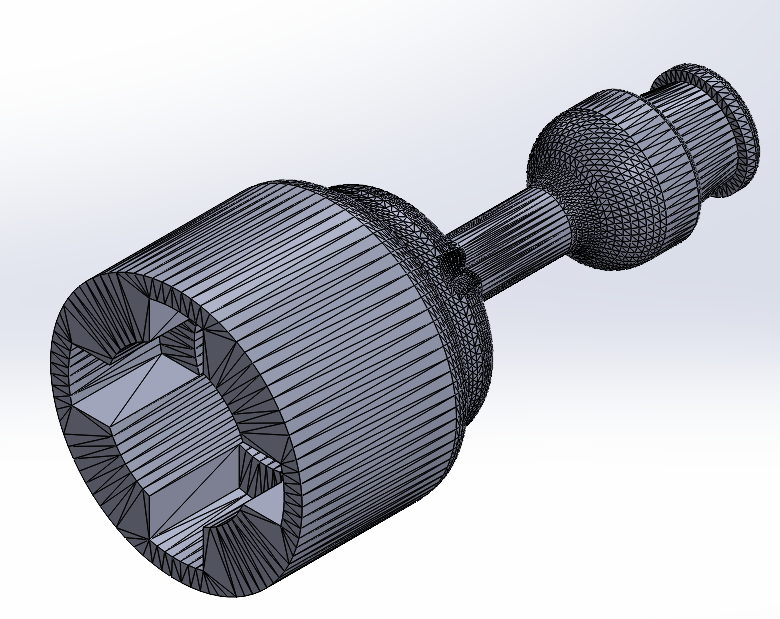
S1 File. Drop dispenser plunger design file.


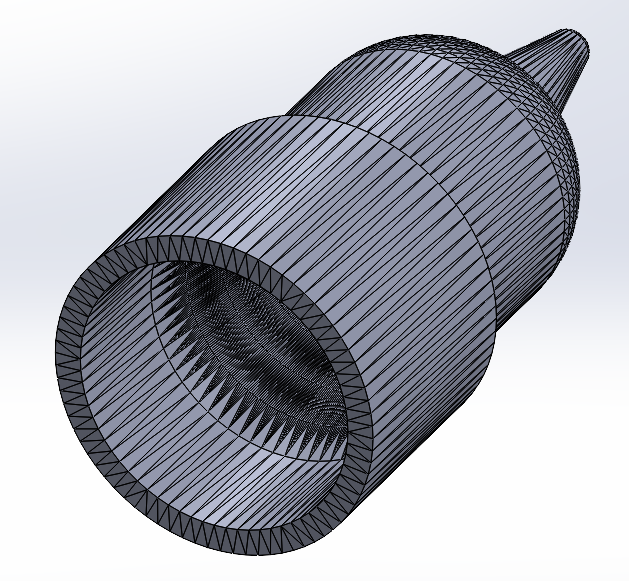
S2 File. Drop dispenser liquid holders design file.
